# Bronchopulmonary dysplasia prediction models: a systematic review and meta-analysis with validation

**DOI:** 10.1038/s41390-022-02451-8

**Published:** 2023-01-09

**Authors:** T’ng Chang Kwok, Natalie Batey, Ka Ling Luu, Andrew Prayle, Don Sharkey

**Affiliations:** 1 Centre for Perinatal Research, School of Medicine, Queen’s Medical Centre, University of Nottingham, E Floor, East Block, Nottingham, NG7 2UH UK; 2 Nottingham Neonatal Service, Queen’s Medical Centre, Nottingham University Hospitals NHS Trust, Nottingham, NG7 2UH UK; 3 Nottingham Maternity Service, Queen’s Medical Centre, Nottingham University Hospitals NHS Trust, Nottingham, NG7 2UH UK; 4 NIHR Nottingham Biomedical Research Centre, Queen’s Medical Centre, Nottingham University Hospitals NHS Trust, C Floor, South Block, Derby Road, Nottingham, NG7 2UH UK

## Abstract

**Abstract:**

Prediction models could identify infants at the greatest risk of bronchopulmonary dysplasia (BPD) and allow targeted preventative strategies. We performed a systematic review and meta-analysis with external validation of identified models. Studies using predictors available before day 14 of life to predict BPD in very preterm infants were included. Two reviewers assessed 7628 studies for eligibility. Meta-analysis of externally validated models was followed by validation using 62,864 very preterm infants in England and Wales. A total of 64 studies using 53 prediction models were included totalling 274,407 infants (range 32–156,587/study). In all, 35 (55%) studies predated 2010; 39 (61%) were single-centre studies. A total of 97% of studies had a high risk of bias, especially in the analysis domain. Following meta-analysis of 22 BPD and 11 BPD/death composite externally validated models, Laughon’s day one model was the most promising in predicting BPD and death (C-statistic 0.76 (95% CI 0.70–0.81) and good calibration). Six models were externally validated in our cohort with C-statistics between 0.70 and 0.90 but with poor calibration. Few BPD prediction models were developed with contemporary populations, underwent external validation, or had calibration and impact analyses. Contemporary, validated, and dynamic prediction models are needed for targeted preventative strategies.

**Impact:**

- This review aims to provide a comprehensive assessment of all BPD prediction models developed to address the uncertainty of which model is sufficiently valid and generalisable for use in clinical practice and research.
- Published BPD prediction models are mostly outdated, single centre and lack external validation.
- Laughon’s 2011 model is the most promising but more robust models, using contemporary data with external validation are needed to support better treatments.

## Introduction

Bronchopulmonary dysplasia (BPD), one of the most common and complex neonatal conditions,^1^ continues to increase and affects approximately 28,000 and 18,000 babies annually in Europe^2^ and the US,^3^ respectively. Preterm infants with BPD have significant long-term respiratory and neurodevelopmental complications into adulthood,^4^ including abnormal lung function^5^ and poor school performance.^4^

There is a myriad of trials with at least 24 Cochrane reviews looking at BPD preventative interventions, including postnatal corticosteroids. However, their benefit in preventing BPD may not outweigh the significant side effects, including gastrointestinal perforation and neurodevelopmental impairment.^6,7^ This demonstrates the complexity of BPD management in balancing the risk of significant long-term morbidity from BPD with that of exposure to potentially harmful treatments.^8^

BPD prediction models aim to provide a personalised risk approach in identifying high-risk very preterm infants for timely preventative treatments. Despite numerous models being developed, none are used routinely in clinical practice. This review aims to provide a comprehensive assessment of all BPD prediction models developed to address the clinical uncertainty of which predictive model is sufficiently valid and generalisable for clinical and research use. Secondly, we will validate eligible models in a large national contemporaneous cohort of very preterm infants.

## Material and methods

### Systematic review

There was no deviation from the protocol published in PROSPERO.^9^ Standard Cochrane Neonatal and Prognosis Methods Group methodologies were used.

#### Inclusion criteria

Cohort, case–control, and randomised controlled trials used in developing or validating the prediction models were included. Very preterm infants born before 32 weeks of gestational age (GA) and less than 2 weeks old at the time of BPD prediction were included. This ensures the clinical applicability and timeliness of the prediction models to support clinical decision making on preventative treatments. Studies that used non-universally accessible predictors such as pulmonary function tests, ultrasonography and biomarkers were excluded. BPD was defined as a respiratory support requirement at either 28 days of age or 36 weeks of corrected gestational age (CGA).^10^ The composite outcome of BPD and death before discharge was included as a secondary outcome.

#### Search methods

Standard Cochrane Neonatal^11^ and prognostic study search filters^12^ were used. “Bronchopulmonary dysplasia OR BPD OR chronic lung disease OR CLD” search terms were used to search the CENTRAL, Ovid MEDLINE, CINAHL, EMBASE and Scopus databases until 13/08/2021 (Appendix 1).

#### Data collection

Two reviewers (T.C.K., N.B. or K.L.L.) independently screened the title and abstract as well as full-text reports for inclusion before independently extracting data and assessing the risk of bias using the PROBAST tool^13,14^ (Appendix 2). These were done using a web-based tool CADIMA.^15^ Any disagreement was resolved by discussion.

#### Prediction model performance measure

Discrimination (C-statistics), calibration (Observed:Expected ratio (O:E ratio)) and classification (net benefit analysis) measures were extracted alongside their uncertainties.

#### Missing data

Study authors were contacted to obtain any missing data. Failing that, missing performance measures were approximated using the methodology proposed by Debray et al.^16^ and R statistical package “metamisc“.^17^

#### Meta-analysis

Meta-analysis of the performance measures, using the random-effects approach and R statistical package “metafor“,^18^ was performed for externally validated models. Sensitivity analysis was performed by excluding studies with an overall high risk of bias. We pre-specified that we would assess the source of heterogeneity^16^ and reporting deficiencies^19^ if more than ten studies were included.

#### Conclusions

The adapted Grades of Recommendation, Assessment, Development and Evaluation (GRADE) framework^20^ was used to assess the certainty of the evidence.

### External validation of eligible models

#### Study design

A population-based retrospective cohort study from the UK National Neonatal Research Database (NNRD)^21^ was used to externally validate BPD prediction models identified in the systematic review. We included all very preterm infants admitted to 185 neonatal units in England and Wales from January 2010 to December 2017. This encompasses over 90% of English neonatal units in 2010, with full coverage in England and Wales in 2012 and 2014 respectively. Infants with birthweight *z* score below –4 or above 4 were excluded as they were likely erroneous entries. Further details of the data items extracted are found within the National Neonatal Dataset^21^ and Appendix 3. Ethical approval was granted by the Sheffield Research Ethics Committee (REC reference 19/YH/0115).

#### Statistical analysis

Data extraction and statistical analysis were done using STATA/SE version 16 (StataCorp) and R version 4 (R Core Team). Summary statistics (median, interquartile range and percentages) were used to describe the data. Missing data were imputed five times using Multivariate Imputation by Chained Equations.^22^ Model performances were assessed in three domains: discrimination (C-statistics), calibration (calibration plot and O:E ratio) and utility measure (decision curve analysis).

## Results

### Systematic review

#### Literature search

**Fig. 1:**
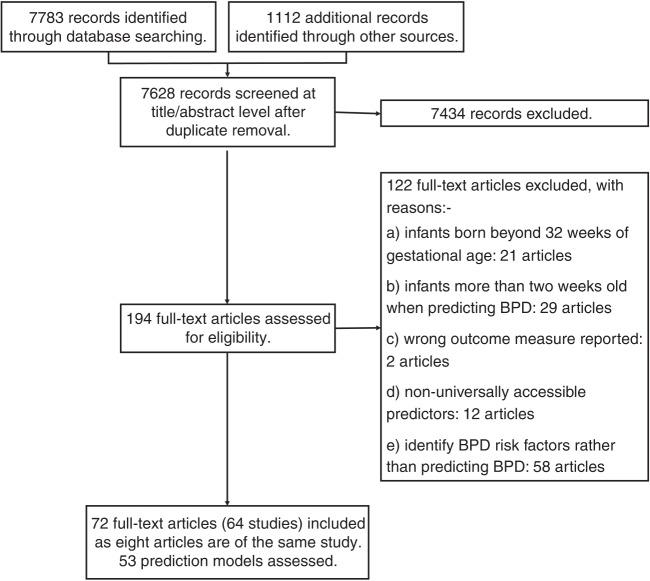
Flow diagram of the systematic review. Flow diagram of literature search and included studies.

Of the 7628 potentially eligible studies identified, 194 full-text articles were screened with 122 articles excluded as studies identified risk factors rather than developing prediction models (48%), predictors available after 2 weeks of age (24%), infants above 32 weeks GA at birth (17%), non-universally accessible predictors (10%) or wrong outcome measure reported (2%). Data were extracted from the 72 full-text articles (Appendix 4), encompassing 64 studies and 53 BPD prediction models (Fig. 1).

#### Description of included studies

Of the 64 included studies, 31 were BPD prediction model development studies, 20 were validation studies, and 13 were development and external validation studies. Fifty-five of the studies were cohort studies; five were randomised controlled trials; two used a combination of randomised control trials and cohort studies with one case–control study and another with an unreported study design. Twenty-six studies were performed in North America, 14 in Europe, 13 in Asia, 5 in South America and Australia/New Zealand each and 1 study was carried out worldwide. Twenty studies developed and validated BPD prediction models based on infants born before 2000, with a further 15 studies using infants born between 2000 and 2010. The 64 included studies recruited 274,407 (range 32 and 156,587) infants, with the majority (50 studies) recruiting less than 1000 infants. A total of 39 (61%) studies were conducted in a single centre. Forty-seven studies used BPD as their outcome, while 14 studies used a BPD/death composite outcome, with 3 further studies reporting both BPD and BPD/death composite outcomes. Thirty-one studies defined BPD at 36 weeks CGA, while 22 studies used the timepoint of 28 days old. Six studies defined BPD using both timepoints. Five studies did not report how BPD was defined (Table 1).

**Table 1:** Characteristics of 64 included studies.

Study	Country/region	Number of centres	Data collection period	Study design	Number of infants	Number of BPD	Number of deaths	Gestation	Birthweight (g)	Outcome
*Development only*										
Cohen 1983	USA	1	1978–1981	Cohort	69	42	NR	NR	NR	BPD 28 d
Palta 1990	USA	5	NR	Cohort	42	36	NR	NR	1042 ± 267	BPD 30 d
Parker 1992	USA	1	1976–1985 (D) 1986–1990 (V)	Cohort	1500 (D) 875 (V)	252 (D) 288 (V)	328 (D) 139 (V)	29.6 ± 2.6 (D) 28.7 ± 2.9 (V)	1113 ± 261 (D) 1066 ± 278 (V)	BPD 28 d and death/BPD 28 d
Corcoran 1993	UK	1	1980–1990	Cohort	412	140	115	29.7 ± 2.8	1345 ± 445	BPD 28 d
Gortner 1996	Germany	1	1985–1992	Case–control	152	76	48	29.2 ± 2.0	1139 ± 249	BPD 28 d
Ryan 1996	UK	1	1991–1992 (D) 1993 (V)	Cohort	204 (D) 47 (V)	**BPD 28** **d** 85 (D) NR (V) **BPD 36** **w** 51 (D) NR (V)	7 (D) NR (V)	27.5 ± 1.5 (D) NR (V)	1283 ± 327 (D) NR (V)	BPD 28 d and BPD 36 w
Groves 2004	NZ	1	1998–2000	Cohort	290	60	54	27.9 ± 8.4	863 ± 626	Death/BPD 36 w
Cunha 2005	Brazil	1	2000–2002	Cohort	86	45	NR	29.0 ± 2.3	1029 ± 222	BPD 28 d
Choi 2006	Korea	1	2000–2005	Cohort	81	48	NR	28.1 ± 1.7	1051 ± 233	BPD 28 d
Henderson-Smart 2006	Australia/NZ	25	1998–1999 (D) 2000–2001 (V)	Cohort	5599 (D) 5854 (V)	1235 (D) 1475 (V)	NR	28.7 ± 2.2 (D) 28.7 ± 2.2 (V)	1233 ± 404 (D) 1235 ± 408 (V)	BPD 36 w
Ambalavanan 2008	USA	16	2001–2003	RCT	420	151	202	26.0 ± 2.0	839 ± 262	Death/BPD 36 w
Gottipati 2012	USA	1	2002–2007	Cohort	417	NR	NR	NR	NR	BPD (NR)
Roth-Kleiner 2012	Switzerland	1	1998–2007	Cohort	936	NR	NR	NR	NR	BPD (NR)
Chock 2014	USA	1	2006–2010	Cohort	187	73	12	27.6 ± 2.0	1005 ± 260	Death/BPD 36 w
Yang 2014	Korea	1	2003–2010	Cohort	261	66	0	30.6 ± 2.4	1549 ± 487	BPD 28 d
Ochab 2016	Poland	1	NR	NR	109	46	NR	NR	NR	BPD 28 d
Wai 2016	USA	25	2010–2013	RCT	495	283	53	25.2 ± 1.2	700 ± 165	Death/BPD 36 w
Kim 2017	Korea	1	2008–2014	Cohort	304	110	13	28.3 ± 2.3	1032 ± 276	Death/BPD 36 w
Beltempo 2018	Canada	30	2010–2015	Cohort	9240	2959	1277	26.7	925 ± 251	BPD 36 w
Boghossian 2018	USA/Puerto Rico	852	2006–2014	Cohort	156,587	NR	NR	NR	NR	BPD 36 w
Hunt 2018	UK	1	2012–2017	Cohort	432	228	7	NR	NR	BPD 28 d
Sullivan 2018	USA	2	2009–2015	Cohort	778	186	48	28.0 ± 2.8	1029 ± 298	BPD 36 w
Fairchild 2019	USA	1	2009–2014	Cohort	502	172	15	27.3 ± 3.0	1023 ± 335	BPD 36 w
Sun 2019	China	1	2015–2018	Cohort	296	144	0	29.9 ± 1.6	1417 ± 328	BPD 28 d
Valenzuela-Stutman 2019	South America	15	2001–2015	Cohort	16,407	2580	3938	29 ± 2.9	1099 ± 275	BPD 36 w and death/BPD 36 w
Dylag 2020	USA	6	2011–2014	Cohort	704	**BPD 28** **d** 414 **BPD 36** **w** 276	0	26.7 ± 1.4	922 ± 229	BPD 28 d
Shah 2020	USA	1	2006–2016	Cohort	730	343	139	27 ± 2	867 ± 198	Death/BPD 36 w
Sharma 2020	USA	1	2011–2017	Cohort	263	155	16	25.2 ± 1.4	805 ± 195	BPD 36 w
Vaid 2020	USA	1	2005–2018	Cohort	1832	NR	NR	NR	NR	BPD (NR)
Shim 2021	Korea	66	2013–2016 (D) 2017–2017 (V)	Cohort	4600 (D) 1740 (V)	**BPD 28** **d** 2583 (D) 1003 (V) **BPD 36** **w** 1370 (D) 463 (V)	1053 (D) 280 (V)	28.7 ± 2.6 (D) 28.8 ± 2.6 (V)	1119 ± 264 (D) 1127 ± 260 (V)	BPD 28 d and BPD 36 w
Ushida 2021	Japan	200	2006–2015	Cohort	31,157	7504	1958	27.8 ± 2.5	973 ± 299	BPD 36 w
*Development and validation*										
Ryan 1994	UK	2	1988–1989	Cohort	166 (D) 133 (V)	47 (D) 59 (V)	NR	29 ± 2.6 (D) 30 ± 3.1 (V)	1043 ± 189 (D) 1056 ± 177 (V)	BPD 28 d
Rozycki 1996	USA	1	1987–1989 (D) 1990–1991 (V)	Cohort	**14** **d model** 116 (D) 61 (V) **8** **h model** 698 (D)	**14** **d model** 38 (D) 34 (V) **8** **h model** 44 (D)	NR	**14** **d model** 26.7 ± 2.0 (D) NR (V) **8** **h mode** 29.7 ± 2.2 (D)	**14** **d model** 911 ± 227 (D) NR (V) **8** **h model** 1352 ± 478 (D)	BPD 28 d
Romagnoli 1998	Italy	1	1989–1991 (D) 1993–1996 (V)	Cohort	50 (D) 149 (V)	28 (D) 82 (V)	NR	28.4 ± 2.1 (D) 28.7 ± 2.4 (V)	893 ± 206 (D) 931 ± 208 (V)	BPD 28 d
Yoder 1999	USA	1 (D) 3 (V)	1990–1992 (D) 1993–1995 (V)	Cohort	48 (D) 110 (V)	15 (D) 33 (V)	NR	27.0 ± 2.0 (D) 26.5 ± 2.1 (V)	897 ± 243 (D) 905 ± 222 (V)	Death/BPD 36 w
Chien 2002	Canada	17	1996–1997	Cohort	4226	NR	NR	29.0 ± 2.0	1390 ± 457	BPD 36 w
Kim 2005	Korea	1	1997–1999 (D) 2000–2001 (V)	Cohort	197 (D) 107 (V)	30 (D) 9 (V)	34 (D) 11 (V)	28.2 ± 1.9 (D) 28.5 ± 1.9 (V)	1043 ± 263 (D) 1095 ± 270 (V)	BPD 36 w
Bhering 2007	Brazil	1	1998–2003 (D) 2003–2005 (V)	Cohort	247 (D) 61 (V)	68 (D) NR (V)	5 (D) NR (V)	29.1 ± 2.4 (D) NR (V)	1083 ± 237 (D) NR (V)	BPD 28 d
May 2007	UK	1	1995–1998 (D) 2004–2005 (V)	RCT (D) Cohort (V)	136 (D) 75 (V)	**BPD 28** **d** 82 (D) 32 (V) **BPD 36** **w** 38 (D) 22 (V)	NR	27.7 ± 2.0 (D) 29.3 ± 2.6 (V)	1017 ± 246 (D) 1245 ± 424 (V)	BPD 28 d and BPD 36 w
Laughon 2011	USA	17	2000–2004	RCT	3629 (D) 1777 (V)	1943 (D) 1215 (V)	468 (D) 210 (V)	26.7 ± 1.9 (D) 25.7 ± 1.1 (V)	897 ± 203 (D) 830 ± 175 (V)	Death/BPD 28 d
Gursoy 2014	Turkey	1	2006–2009 (D) 2012 (V)	Cohort	652 (D) 172 (V)	150 (D) 54 (V)	NR	29.4 ± 1.9 (D) 28.9 ± 2.3 (V)	1218 ± 220 (D) 1102 ± 251 (V)	BPD 28 d
Anand 2015	USA	2	NR	Cohort	49 (D) 46 (V)	16 (D) NR (V)	NR	NR	NR	BPD 28 d
Mistry 2020	Australia	NR	NR	Cohort	NR	NR	NR	NR	NR	BPD (NR)
Baud 2021	France	21	2008–2014	RCT	523	125	107	26.4 ± 0.8	854 ± 170	Death/BPD 36 w
*Validation only*										
Fowlie 1998	UK	6	1988–1990	Cohort	398	**BPD 28** **d** 75 **BPD 36** **w** 31	81	29.8 ± 2.5	1065 ± 186	BPD 28 d and BPD 36 w
Hentschel 1998	Germany	1	1991–1993	Cohort	188	**BPD 28** **d** 61 **BPD 36** **w** 45	30	28.6 ± 0.3	1101 ± 281	BPD 28 d and BPD 36 w
Schroeder 1998	Germany	1	1985–1992	Cohort	103	59	NR	28.5 ± 1.9	1000 ± 153	BPD 28 d
Srisuparp 2003	USA	1	1996–1997	Cohort	138	47	24	27.6 ± 2.4	995 ± 247	BPD 36 w
Thowfique 2010	Singapore	1	2006–2007	Cohort	388	59	40	28.7 ± 3	1029 ± 251	BPD (NR)
Carvalho 2011	Brazil	2	2002–2009	Cohort	86	20	18	28.3 ± 1.8	851 ± 233	BPD 36 w
Onland 2013	Worldwide	85	1986–2004	10 RCTs	3229	1094^a^	582^a^	27.3 ± 3.8	989 ± 315	BPD 36 w and death/BPD 36 w
Truog 2014	USA	1	2008–2010	Cohort	158	115	15	NR	NR	Death/BPD 36 w
Sullivan 2016	USA	1	2004–2014	Cohort	566	98	51	28.6 ± 2.9	NR	BPD 36 w
Ozcan 2017	Turkey	1	2011–2012	Cohort	246	28	32	29.2 ± 2.15	1323 ± 331	BPD 36 w
Gulliver 2018	USA	1	2010–2016	Cohort	622	223	61	27.0 ± 1.9	963 ± 301	Death/BPD 36 w
Vasquez 2018	Colombia	2	2010–2016	Cohort	335	68	NR	31 ± 1.5	1328 ± 328	BPD 28 d
Jung 2019	Korea	1	2010–2014	Cohort	138	57	17	25.9 ± 1.3	780 ± 225	Death/sev BPD 36 w
Lee 2019	Korea	67	2013–2016	Cohort	6938	1916	957	28.3 ± 2.4	1059 ± 283	BPD 36 w
Baker 2020	Australia	2	2016–2017	Cohort	187	72 (**sev BPD**)	18	26.6 ± 1.5	872 ± 178	Death/sev BPD 28 d
Bhattacharjee 2020	USA	1	2012–2013	Cohort	69	31	5	24.75 ± 1.5	722 ± 160	Sev BPD 36 w
Sotodate 2020	Japan	1	2010–2017	Cohort	171	74	19	25.5 ± 1.6	741.3 ± 208.2	BPD 36 w
Steocklin 2020	Australia	1	2017–2018	Cohort	32	NR	NR	26.2 ± 1.0	NR	BPD 36 w
Alonso 2021	Spain	1	2013–2020	Cohort	202	**BPD 28** **d** 58 **BPD 36** **w** 21	23	29.5 ± 2.1	1142 ± 256	BPD 28 d and BPD 36 w
Rysavy 2021	USA	14 (RCT) 32 (cohort)	1996–1997 (RCT) 2016–2018 (cohort)	RCT/cohort	807 (RCT) 2370 (cohort)	356 (RCT) 1417 death/BPD (cohort)	114 (RCT) 1417 death/BPD (cohort)	25.8 ± 1.8 (RCT) NR (cohort)	770 ± 136 (RCT) NR (cohort)	Death/BPD 36 w

*NR* not reported, *D* derivation, *V* validation, *RCT* randomised controlled trials, *Sev* severe.

^a^Data obtained from the original study protocol (Cools et al.^23^).

A total of 70% of the 44 derivation studies used logistic regression to develop the BPD prediction tool, with 11% used univariate analysis; 5% used clinical consensus as well as a combination of logistic regression and classification and tree analysis (CART) respectively; and 2% used CART, gradient boosting, Bayesian network and a combination of logistic regression and support vector machine, respectively. Complete case analysis was used in 41% of the included derivation studies, while handling of missing data was not reported in the remaining 59%. Internal and external validation was done in 25% and 30% of the studies, respectively. Validation was not done in the remaining 45% of studies. A total of 75% of the studies assessed discrimination using C-statistics. In contrast, only 16% of the studies evaluated calibration using the goodness of fit (5 studies), calibration plot (1 study) and O:E ratio (1 study). Of the 44 models, ten (23%), eight (18%) and four (9%) models provided a formula, score chart and nomogram respectively. Only two (5%) models provided an online calculator (Table 2).

**Table 2:** Methodology used by the derivation studies.

Study	Model development					Model validation		
	Approach	Continuous predictor	Missing value	Predictor selection	Presentation	Approach	Discrimination	Calibration
Cohen 1983	Clinical consensus	Categorical	Complete case	Clinical consensus	NR	Same cohort	NR	None
Palta 1990	Clinical consensus	Kept linear	NR	Clinical consensus	Formula	Same cohort	NR	None
Parker 1992	Regression	Kept linear	Complete case and Replace mean	Univariate → stepwise	Formula	Bootstrapping/temporal (year)	NR	O:E ratio
Corcoran 1993	Regression	Categorical	Complete case	Univariate → stepwise	Formula	Random split	NR	None
Ryan 1994	Regression	Kept linear	NR	Univariate → stepwise	Formula	New dataset	ROC	None
Gortner 1996	Regression	Kept linear	NR	Univariate → stepwise	Formula	Same cohort	NR	None
Rozycki 1996	Regression	Categorical	Complete case	Univariate → stepwise	Nomogram	New dataset	NR	None
Ryan 1996	Regression	Kept linear	NR	Univariate → stepwise	Formula	Temporal (year)	ROC	None
Romagnoli 1998	Regression	Categorical	NR	Univariate analysis	Formula	New dataset	ROC	None
Yoder 1999	Regression	Categorical	NR	Univariate analysis	Score chart	New dataset	ROC	None
Chien 2002	Regression	Kept linear	Complete case	NR	NR	Same cohort	ROC	Goodness of fit
Groves 2004	Univariate analysis	Kept linear	NR	Stepwise selection	NR	Same cohort	ROC	None
Cunha 2005	Regression	Categorical	NR	Univariate → stepwise	Score chart	Same cohort	NR	None
Kim 2005	Regression	Categorical	Complete case	Univariate → stepwise	Score chart	New dataset	ROC	None
Choi 2006	Regression	Categorical	NR	Univariate → stepwise	Score chart	Same cohort	ROC	None
Henderson-Smart 2006	Regression	Categorical	Complete case	Univariate → stepwise	Formula	Temporal (year)	ROC	Goodness of fit
Bhering 2007	Regression	Categorical	Complete case	Stepwise selection	Score chart	New dataset	ROC	Goodness of fit
May 2007	Univariate analysis	Kept linear	NR	No selection	NR	New dataset	ROC	None
Ambalavanan 2008	Regression and CART	Kept linear	Complete case	Stepwise selection	Nomogram	Cross-validation	ROC	None
Laughon 2011	Regression	Kept linear	NR	Univariate → stepwise	Formula/online tool	New dataset	ROC	None
Gottipati 2012	Regression	Kept linear	NR	NR	Score chart	Same cohort	NR	None
Roth-Kleiner 2012	Regression	Categorical	NR	Stepwise selection	Score chart	Cross-validation	ROC	None
Chock 2014	Regression and CART	Kept linear	NR	NR	Nomogram	Same cohort	ROC	None
Gursoy 2014	Regression	Categorical	NR	Univariate → stepwise	Score chart	New dataset	ROC	Goodness of fit
Yang 2014	Univariate analysis	Kept linear	NR	No selection	NR	Same cohort	ROC	None
Anand 2015	Bayesian Network	Categorical	NR	NR	NR	New dataset	ROC	None
Ochab 2016	Regression and SVM	Kept linear	NR	Stepwise selection	NR	Cross-validation	NR	None
Wai 2016	Regression	Kept linear	Complete case	Univariate → stepwise	NR	Same cohort	ROC	None
Kim 2017	Regression	Kept linear	NR	Univariate analysis	NR	Same cohort	NR	None
Beltempo 2018	Regression	Categorical	Complete case	No selection	NR	Same cohort	ROC	None
Boghossian 2018	Regression	Categorical	NR	No selection	NR	Random split	ROC	None
Hunt 2018	Univariate analysis	Categorical	NR	No selection	NR	Same cohort	ROC	None
Sullivan 2018	Regression	Transformation	NR	No selection	NR	Bootstrapping	ROC	None
Fairchild 2019	Regression	Kept linear	NR	Stepwise selection	NR	Same cohort	ROC	None
Sun 2019	Univariate analysis	Kept linear	Complete case	No selection	NR	Same cohort	ROC	None
Valenzuela-Stutman 2019	Regression	Kept linear	NR	Stepwise selection	NR	Random split	ROC	None
Dylag 2020	Regression	Kept linear	Complete case	Stepwise selection	NR	Random split	ROC	None
Mistry 2020	Regression	Kept linear	NR	NR	NR	Same cohort	ROC	None
Shah 2020	Regression	Kept linear	Complete case	A priori knowledge	NR	Same cohort	ROC	None
Sharma 2020	CART	Kept linear	Complete case	CART	Nomogram	Same cohort	ROC	None
Vaid 2020	Gradient boosting	NR	Complete case	NR	NR	Cross-validation	ROC	None
Baud 2021	Regression	Kept linear	NR	Stepwise selection	Formula	Same cohort	ROC	Goodness of fit
Shim 2021	Regression	Kept linear	Complete case	Stepwise selection	Formula	Temporal (year)	NR	None
Ushida 2021	Regression	Kept linear	Complete case	Stepwise selection	Formula/online tool	Random split	ROC	Calibration plot

*NR* not reported, *CART* classification and regression tree, *SVM* support vector machine, *ROC* receiver operating characteristics curve, *O:E ratio* observed:expected ratio.

Of the 53 BPD prediction models identified, 19 used predictors available within 24 hours of age, while 20 and six models relied on predictors available between 2 and 7 days and above 7 days of age, respectively. Seven models used predictors available at various timepoints while the timepoints were unavailable for one model. The BPD prediction models considered a median of 14 predictors before using a median of five predictors in the final models. The five most used predictors were GA, birthweight, the fraction of inspired oxygen (FiO_2_), sex and invasive ventilation requirement, which were used in 33–69% of models (Appendix 5).

#### Risk of bias

The majority of the studies were assessed to have a low risk of bias for the three domains of participants (84%), predictors (92%) and outcome (89%). A total of 60 (94%) studies were assessed to have a high risk of bias in the analysis domain based on the PROBAST tool^13^ due to various reasons including calibration not assessed (55 studies (86%)); small sample size (37 studies (58%)); inappropriate handling of missing data (21 studies (33%)); lack of internal/external validation (9 studies (14%)); inappropriate selection approach for predictors (6 studies (9%)); and inappropriate handling of continuous predictors (2 studies (3%)).

**Fig. 2:**
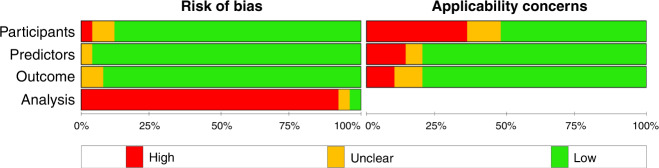
Risk of bias assessment. Summary of risk of bias assessments for included studies based on the PROBAST tool.^13^

Twenty-one studies (33%) had high applicability concerns in the participant’s domain as they targeted a specific group of very preterm infants, usually infants at a higher risk of BPD (for example, ventilated infants only in 17 studies (27%)). Although universally accessible, predictors used in ten studies (16%) may not be routinely collected. Eight studies (13%) used BPD definitions that deviated against the current consensus^10^ (Fig. 2 and Appendix 6).

#### Discrimination

The C-statistics of the included prediction models ranged from 0.52 to 0.95 in the external validation studies with better performance in models using predictors beyond 7 days of age. Meta-analysis could only be done on 22 (50%) and 11 (35%) models for BPD and BPD/death composite outcomes, respectively, as the remaining 22 and 20 models were only validated in one study. The C-statistics confidence intervals (CI) were wide due to the small number of studies in each meta-analysis. The five models with CI above 0.5 for BPD from the meta-analysis were CRIB I,^24^ CRIB II^25^ as well as Valenzuela-Stutman 2019^26^ (Birth, day 3 and 14 models). Similarly, for the BPD/death composite, the five models with CI above 0.5 from the meta-analysis were Laughon 2011^27^ (day 1, 3, 7 and 14 models) and Valenzuela-Stutman 2019^26^ (day 14 model) (Appendix 7). Meta-analysis for the Valenzuela-Stutman 2019 models^26^ could only be performed after including validation findings from our cohort study.

#### Calibration

The O:E ratio was reported in four external validation studies^28–31^ evaluating six prediction models (Rozycki 1996,^28^ Parker 1992^29^ and Laughon 2011^27^ (day 1, 3, 7 and 14 models)) with considerable variation in the O:E ratio among the included models. Meta-analysis of the O:E ratio could only be done on one model (Laughon 2011^27^ (day 1)) with an O:E ratio of 0.96 (95% CI 0.85–0.99) (Appendix 8).

The calibration plot was reported in three studies,^30–32^ assessing six models (Palta 1990,^33^ Sinkin 1990,^34^ Ryan 1996,^35^ Kim 2005^36^ as well as Laughon 2011^27^ (day 1 and 3 models) (Appendix 9).

#### Classification

No studies reported net benefit or decision curve analyses.

#### Heterogeneity and reporting deficiencies

The wide confidence and prediction intervals demonstrated heterogeneity amongst the external validation studies. As there were less than ten validation studies in a meta-analysis, subgroup analysis and funnel plots were not performed to explore the source of heterogeneity. Sensitivity analysis was not performed as all studies had an overall high risk of bias except for two studies.^30,37^

#### Summary of findings

Due to the lack of validation studies, a conclusion could only be made for one model Laughon 2011.^27^ There was a low quality of evidence to show the discrimination and calibration performances of the Laughon 2011^27^ model in predicting the BPD/death composite outcome using predictors at day 1 of age with a C-statistic of 0.76 (95% CI 0.70–0.81) and O:E ratio of 0.96 (95% CI 0.85–0.99). The evidence was downgraded by two levels due to study limitation (variation in the BPD definition used and some studies recruiting high-risk infants only (such as invasively ventilated infants)), as well as inconsistency (heterogeneity and a small number of external validation studies).

### External validation

#### Patient cohort

After exclusions (Appendix 10), 62,864 very preterm infants were included (Appendix 11). A total of 17,775 (31%) infants developed BPD while 5718 (9%) infants died before discharge from the neonatal unit.

#### Model performance

**Fig. 3:**
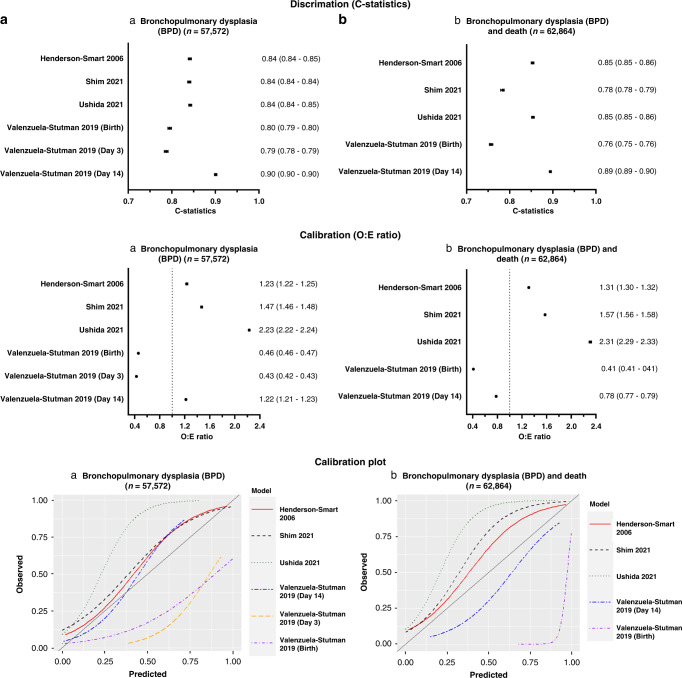
Model performance of the externally validated prediction models. Discrimination (C-statistics) and calibration (O:E ratio and calibration plots) characteristics of prediction models externally validated using a retrospective cohort for **a** bronchopulmonary dysplasia (BPD) (*n* = 57,572) and **b** composite BPD and death (*n* = 62,864).

We were able to externally validate six prediction models (Henderson-Smart 2006,^38^ Valenzuela-Stutman 2019^26^ (day 1, 3 and 14 models), Shim 2021^39^ and Ushida 2021^40^) in our retrospective cohort. The variables in the remaining models were not available in our cohort. The discrimination (C-statistics) and calibration (O:E ratio and calibration plot) (Fig. 3) performances were variable among the models. Although the models displayed fair to good discrimination with C-statistics of 0.70–0.90, they had poor calibration as indicated by the calibration plot and O:E ratio between 0.39 and 2.31. The Valenzuela-Stutman 2019 models^26^ appear to overestimate the predicted risk, whereas the remaining three models (Henderson-Smart 2006,^38^ Shim 2021^39^ and Ushida 2021^40^) tend to underestimate the predicted risk. Of the six externally validated models, four models (Henderson-Smart 2006,^38^ Valenzuela-Stutman 2019^26^ (day 14 models), Shim 2021^39^ and Ushida 2021^40^) indicated superior net benefit across a reasonable range of threshold probabilities of 30–60% in deciding postnatal corticosteroid treatment in the decision curve analysis (Appendix 12). The threshold probabilities used were identified in a meta-regression of 20 randomised controlled trials.^8^

## Discussion

Our study is an update to the systematic review carried out nearly a decade ago,^32^ with a further 27 prediction models identified since the last review. Our systematic review identified 64 studies that developed and/or validated 53 BPD prediction models with meta-analysis carried out on 22 models. Due to the lack of external validation studies, we could not identify a prediction model for routine clinical use. Further external validation, including assessment of both discrimination and calibration performances in a population similar to that whereby the model will be used, is needed before any model could be adopted in clinical practice. However, the most promising prediction model that could be considered based on our meta-analysis was Laughon 2011^27^ in predicting the BPD/death composite outcome using predictors at day one of age. Further re-calibration of the model based on the local population of interest, with re-assessment of its performance in subsequent external validation studies (if re-calibrated), may be needed before being used in clinical practice.

We have also externally validated six prediction models^26,38–40^ in our retrospective population-based cohort study as variables in the remaining models were not available in our cohort. Although they have fair to good discrimination, they were not well calibrated in our cohort. To be useful, prediction tools need to be generalisable to current datasets highlighting the importance of external validation.

### Implications for clinical practice and research

The implementation of BPD prediction models in clinical practice is limited by the lack of external validation of the published models. Less than a third of the identified prediction models were externally validated. Furthermore, half of the externally validated models were only validated by one study. This potentially limits the generalisability of the model performance to other infant populations and adoption into clinical practice. There is also a need for continual assessment of the model performance over time to determine if further updates to the model are needed with changes in clinical practice.

#### Sample size

Most external validation studies had small sample sizes or were restricted to specific high-risk infant populations (for example, ventilated infants only). Furthermore, 61% of studies were single centre only. This potentially limits the generalisability of the models. It is recommended that prediction model development studies should have a sufficient sample size of infants with the outcome of interest for the number of candidate predictors used based on recommendations made by Riley et al. 2019,^41^ while validation studies should have at least 100 infants with the outcome.^13^

#### Missing values

The majority of the studies did not report missing data or excluded infants with missing data. A clear description of the handling of missing data should be provided. Complete case analysis should be avoided if possible.^13^

#### Variation in prediction timepoint and outcome definition

Nearly three-quarters of the included prediction models predicted BPD, the remainder predicting the BPD/death composite outcome. As death and BPD are semi-competitive risks, infants who died before 36 weeks CGA may have a higher risk of developing BPD if they had survived until 36 weeks CGA. Hence, the potential predictive information of death should be accounted for in BPD prediction modelling. The included models also made predictions at a variety of timepoints. Therefore, a meta-analysis of the models was difficult and may limit the clinical settings in which the model can be used. It may be sensible for the performance of future prediction models to be externally validated for BPD as well as the BPD/death composite outcome at three prediction timepoints of one, seven days and 14 days of age. These timepoints would allow timely preventative treatment or research recruitment to be targeted to high-risk infants.

#### Predictors

The predictors used in the model should be easily assessed routinely during daily clinical practice and not dependable on clinical practice, such as weight loss and fluid intake. Future prediction models should also be dynamic, accounting for the changing status of the infant over time and clinical trajectory.

Predictor selection based on the traditional stepwise approach or univariable analysis should be avoided, especially in small datasets. Instead, predictor selection based on a priori knowledge or statistical approach not based on prior statistical tests between predictor and outcome (e.g., principal component analysis) may be better methods.^14^

#### Model performance

Both discrimination (C-statistics) and calibration (calibration plot or O:E ratio) performances of the prediction models need to be assessed during external validation. A model with fair to good discrimination may be poorly calibrated.^32^ Hosmer-Lemeshow goodness-of-fit test alone without other calibration measures was found not to be a suitable method to assess calibration as it is sensitive to sample size.^13^ The test is often non-significant (i.e. good calibration) in small datasets while usually significant (i.e., poor calibration) in large datasets. Since the recommendation to assess calibration in the last review nearly a decade ago,^32^ only two further studies^30,31^ assessed calibration using calibration plots or O:E ratios.

An impact analysis was not carried out in any of the identified prediction models to evaluate if the prediction model improved patient outcomes. Decision curve analysis^42^ may be used as an initial screening method to assess the net benefit of using the prediction model before carrying out further impact analysis. Decision curve analysis can be used on the external validation dataset without further data collection.

#### Practicality of model

Prediction models developed should be practical and easy to use at the bedside. Only two published models^27,40^ provided online calculators to allow easy access risk assessment.

Changes in clinical practice and rising BPD rates, potentially makes previously published models outdated affecting their predictive ability. Over half of the published models used data from babies born more than a decade ago. Hence, new models should consider a built-in feature to allow them to learn from future babies and adapt their performance to new practices.

### Strength

The systematic review was carried out based on standard Cochrane methodologies as well as recent recommendations for meta-analysis of prediction models^16^ and risk of bias assessment.^13^ There were no language or date restrictions. The review is anticipated to guide clinicians and researchers in not only developing and/or validating BPD prediction models in very premature infants based on recommendations of the review, but also in identifying the most promising prediction model to be externally validated in their local population.

The use of recent routinely collected clinical information in our external validation study, coupled with its large population coverage, provides an accurate representation of the current neonatal practice in England and Wales. This large cohort of nearly 63,000 very preterm infants, including infants receiving both invasive and non-invasive ventilation, forms an ideal cohort to externally validate and assess BPD prediction models.

### Limitation

Only 6 out of the 53 identified prediction models could be validated in our cohort. Hence, the performance of the remaining models in our cohort was unclear. However, it is crucial that future models should only use predictors that are easily assessed in clinical practice to ensure their successful clinical implementation.

## Conclusion

As preterm infant survival increases, more survivors are diagnosed with BPD along with the long-term respiratory and neurological consequences. Despite almost a doubling in the number of BPD prediction models published over the last decade, most identified in our systemic review are not used in routine clinical practice. This is due to a lack of good quality external validation studies assessing their performance on the local population of interest. Furthermore, calibration of the models is often not appropriately evaluated in most of the models. Models should be externally validated with a subsequent impact analysis before being adopted in clinical practice. Decision curve analysis may be a good screening tool to assess the net benefit of the tool prior to impact analysis.

Our systematic review has also made recommendations for future BPD prediction models including consideration of additional predictors, a more dynamic model accounting for changes in the infant’s condition over time and their trajectory, and the ability to adapt performance with evolving clinical practice. A good quality, well-validated BPD prediction tool is needed to provide personalised preventative treatment and allow targeted trial recruitment to reduce the long-term impact on this vulnerable and expanding population.

## Supplementary information

Supplementary Material

Supporting Document - PRISMA checklist

## Data Availability

All data generated or analysed during the systematic review are included in this published article and its supplementary information files. The data that support the findings of the external validation are available from the National Neonatal Research Database but restrictions apply to the availability of these data, which were used under license for the current study, and so are not publicly available. Data are however available from the authors upon reasonable request and with permission of the Research Ethics Committee and National Neonatal Research Database.
